# Association of Social Isolation and Loneliness With Chronic Low Back Pain Among Older Adults: A Cross-sectional Study From Japan Gerontological Evaluation Study (JAGES)

**DOI:** 10.2188/jea.JE20230127

**Published:** 2024-06-05

**Authors:** Taiji Noguchi, Takaaki Ikeda, Takao Kanai, Masashige Saito, Katsunori Kondo, Tami Saito

**Affiliations:** 1 Department of Social Science, Center for Gerontology and Social Science, Research Institute, National Center for Geriatrics and Gerontology, Aichi, Japan; 2 Japan Society for the Promotion of Science, Tokyo, Japan; 3 Department of Health Policy Science, Graduate School of Medical Science, Yamagata University, Yamagata, Japan; 4 Department of International and Community Oral Health, Tohoku University Graduate School of Dentistry, Sendai, Japan; 5 Tokyo Metropolitan Tama-Hokubu Medical Center, Tokyo, Japan; 6 Department of Psychiatry and Behavioral Sciences, Graduate School of Medical and Dental Sciences, Tokyo Medical and Dental University, Tokyo, Japan; 7 Faculty of Social Welfare, Nihon Fukushi University, Aichi, Japan; 8 Department of Social Preventive Medical Sciences, Center for Preventive Medical Sciences, Chiba University, Chiba, Japan; 9 Department of Gerontological Evaluation, Center for Gerontology and Social Science, Research Institute, National Center for Geriatrics and Gerontology, Aichi, Japan

**Keywords:** chronic pain, loneliness, low back pain, social isolation

## Abstract

**Background:**

Per the biopsychosocial model, pain, especially chronic low back pain, which often presents with nonspecific pain, requires a comprehensive approach involving social factors. However, the association of social factors, including social isolation and loneliness, with this condition remains unclear. This study examined the cross-sectional association of social isolation and loneliness with chronic low back pain among older adults.

**Methods:**

We recruited functionally independent older adults through a mail survey in 2019 from the Japan Gerontological Evaluation Study (JAGES). Chronic low back pain was defined as low back pain lasting more than 3 months. Social isolation was identified based on face-to-face and non-face-to-face interactions (“not isolated,” “isolated tendency,” and “isolated”). Loneliness was assessed using the University of California, Los Angeles Loneliness Scale (“not lonely,” “lonely tendency,” and “lonely”).

**Results:**

Consequently, 21,463 participants were analyzed (mean age: 74.4 years; 51.5% females); 12.6% reported chronic low back pain. Multivariable Poisson regression analysis revealed that loneliness was significantly associated with the likelihood of chronic low back pain; compared with “not lonely”, the prevalence ratio (PR) was 1.14 (95% confidence interval [CI], 1.05–1.25) for “lonely tendency” and 1.40 (95% CI, 1.27–1.54) for “lonely.” Social isolation was not associated; compared with “not isolated,” the PR was 0.96 (95% CI, 0.88–1.05) for “isolated tendency” and 0.99 (95% CI, 0.89–1.10) for “isolated.” A positive multiplicative interaction between social isolation and loneliness for chronic low back pain was found.

**Conclusion:**

Lonelier individuals were more likely to experience chronic low back pain, and those with loneliness and social isolation were synergistically more likely for this condition.

## INTRODUCTION

Low back pain is the most commonly experienced among musculoskeletal pains for all age groups.^1^ The prevalence of low back pain in adults is as high as 38.0%, and it is more likely to be higher in older populations.^2^ Low back pain is associated with the disability onset, as well as causing multidimensional economic impacts through increased burdens on medical care and the social welfare systems.^3^ Therefore, the development of prevention strategies against low back pain is increasingly important.

Pain, including low back pain, is defined as “an unpleasant sensory and emotional experience associated with, or resembling that associate with, actual or potential tissue damage,” and further noted that pain is a personal experience influenced by multiple factors, including physical, psychological, and social factors, rather than only related to organic causes, such as tissue damage or pathoanatomical changes.^4^ This definition has contributed to wider acceptance of biopsychosocial models as approaches to pain,^5^^,^^6^ and low back pain is one of the best-understood conditions regarding the multidimensional influences. Despite a large body of literature on the impact of biological factors (eg, tissue damage or anatomical changes)^7^^,^^8^ and psychological factors (eg, depression, psychological distress, somatization, and tendency to catastrophize),^9^^,^^10^ the influence of social factors on low back pain has not been well understood. Particularly, chronic low back pain is often a nonspecific condition without lower limb neurological disorders or serious underlying diseases.^11^^,^^12^ Therefore, a comprehensive approach based on a biopsychosocial model is warranted to effectively manage chronic low back pain. Since social factors may serve as risk factors for chronic pain and as determinants of health outcomes of individuals with chronic pain,^13^ more attention is required to be directed toward exploring the roles of social factors in the context of low back pain.

Among individual social factors, social isolation and loneliness have been increasingly recognized as important social determinants of health.^14^^–^^16^ Social isolation refers to the quantity of social relationships that result from a decrease in social networks with a reduction in the number of social contacts.^17^ Loneliness is a subjective emotional state that individuals might experience with the existence of a gap between their perceived and desired levels of social relationships.^18^ Although social isolation and loneliness are interrelated, they are not necessarily interchangeable and the relationship has been shown as weak to moderate.^19^^–^^21^ This is because people who are socially connected can still feel lonely and, conversely, socially isolated individuals may not feel lonely.^22^ Social isolation and loneliness have significant negative health consequences, including cardiovascular disease,^15^^,^^23^ infectious diseases,^24^ cognitive declines,^25^^,^^26^ and mortality.^14^^,^^27^^,^^28^

Nonetheless, the association of social isolation and loneliness with low back pain is not well understood. On the one hand, musculoskeletal pain leads to a loss of independence and functional disability through activity limitation, muscle atrophy, and fatigue, with consequent social isolation and loneliness based on chronicity.^29^^,^^30^ On the other hand, social isolation and loneliness may enhance pain perception, resulting in increased rumination and negative thoughts about pain.^31^^,^^32^ However, previous studies have produced inconsistent findings. A study among older adults showed that loneliness was associated prospectively with the likelihood of low back pain.^33^ Additionally, perceived social isolation and loneliness during an infectious epidemic had a positive correlation with low back pain.^34^ Whereas, the Friendship Scale, which assesses loneliness and social support, predicted subsequent disability by low back pain but not pain catastrophizing^35^; according to another study, social isolation was not common among patients with low back pain.^36^

Given the differences in the nature of social isolation and loneliness, it is imperative to examine the effects of both factors on low back pain. Additionally, the interaction effects of these two factors on low back pain are yet to be clarified, which invites further investigations. A lack of social relationships, characterized by social isolation and loneliness, is a part of the social domain in the multidimensional factorial classification of low back pain,^6^ particularly in chronic low back pain wherein nonspecific pain is a common occurrence. However, there is insufficient empirical evidence of the association of social isolation and loneliness with low back pain. Addressing these knowledge gaps can help in a more comprehensive understanding of the social factorial approach to low back pain. Furthermore, considering that chronic low back pain is more common in older age than in adulthood and many of the causes are nonspecific,^37^ focusing attention on social factors for this condition in the older populations is essential.

Accordingly, this study examined the associations of social isolation and loneliness with chronic low back pain among older adults, focusing on the interaction effects of both factors.

## METHODS

### Study participants

This cross-sectional study used data from the Japan Gerontological Evaluation Study (JAGES) 2019. The JAGES is an ongoing cohort study investigating functionally independent older adults aged over 65 years, who are not eligible to receive public long-term care insurance (LTCI) benefits.^38^ The survey was conducted from November 2019 to January 2020 using self-administered questionnaires that were mailed to all eligible residents of 60 municipalities in 24 prefectures (out of 47). We obtained a random sample of residents from the official residence registers in 43 large municipalities and a complete census of residents of the other 17 smaller municipalities; 260,252 individuals returned the questionnaires (response rate: 75.4%). Among them, we excluded those who received the LTCI benefits (*n* = 11,541), those who did not provide informed consent (*n* = 36,702), those without information on sex and/or age (*n* = 79), those aged under 65 years (*n* = 14), and those who lived in municipalities that refused academic cooperation (*n* = 18,586). Thus, 197,509 individuals were included. Each participant was randomly assigned one of the eight questionnaire versions, and 24,342 participants received a survey module on pain. We further excluded individuals who received care in their daily lives without using the LTCI benefits (*n* = 1,397) and those with missing data on this item (*n* = 1,482) to avoid the effect of substantial differences in their levels of activity of daily living (ADL) independence. Finally, 21,463 participants were included.

This study was reviewed and approved by the Ethics Committees at Chiba University (No. 2493) and the National Center for Geriatrics and Gerontology (No. 992-3). All participants were informed that their involvement in the study was voluntary and that returning the questionnaire via mail with the acceptance checkbox marked served as an indication of their consent to participate in the study. All our procedures conformed to the principles embodied in the Declaration of Helsinki.

### Chronic low back pain

Participants were asked the following questions: “During the past year, have you ever had low back pain that lasted more than 1 day, except for pain due to an illness with fever?” (possible responses: “yes” or “no”); “If you had low back pain, how long did it last?” (possible responses: “<1 month,” “1–3 months,” or “≥3 months”). Participants responded by reviewing the low back illustration in eFigure 1. Based on the international definition,^39^ we classified participants who reported experiencing persistent low back pain for at least 3 months as having chronic low back pain, whereas those who did not meet this criterion as not having chronic low back pain.

Participants who experienced pain for any duration were further assessed for pain intensity by the following question: “How would you rate the pain on a scale of 0 to 10, with 0 being no pain and 10 being the worst pain you have ever experienced?” (ie, the Numerical Rating Scale [NRS]). Based on previous research,^40^^,^^41^ we defined those with chronic low back pain and an NRS score of ≥5 as having moderate/severe chronic low back pain; the definition is associated with considerable pain or more in older adults.^42^

The following question was used to assess participants who experienced pain of any duration for having disabilities in activities: “Have you ever experienced difficulty or restrictions in doing your regular activities due to the pain?” (possible responses: “yes” or “no”). We defined those with chronic low back pain and living disability as having chronic low back pain with disability in activities.

### Social isolation

Social isolation was evaluated based on the frequency of social contact outside the family living together, based on previous studies.^27^^,^^43^ Namely, participants were asked regarding the frequency of face-to-face and non-face-to-face (eg, phone calls, e-mails, or mail) contacts with family and relatives living apart or friends. These were measured by seven categories from “almost every day” to “none,” and we converted the response items into the number of monthly contacts, with 4.3 weeks considered as the number of weeks per month. The response items were then coded as none = 0, almost none = 0.1, once or twice a month = 1.5, once a week = 4.3, two or three times a week = 10.8, and up to almost every day = 21.5. All converted responses were summed up and categorized into three groups; based on a previous study,^43^ we defined an isolated condition as contact less than once a week; contact frequency of less than half a week was also further separated in the present study as an isolation tendency: “not isolated” (≥17.2: four or more times a week), “isolated tendency” (4.3–17.1: once to three times a week), and “isolated” (<4.3: less than once a week). The Cronbach’s α = 0.67 for this scale was noted.

### Loneliness

Loneliness was assessed using the three items of the University of California, Los Angeles (UCLA) Loneliness Scale,^44^ Japanese version.^45^ Each of the three items—namely a lack of companionship, feeling left out, and feeling isolated from others—had three possible responses: 1 = “hardly ever,” 2 = “some of the time,” and 3 = “often.” The summed responses generate a score that can range from 3 to 9 points, with higher scores indicating greater loneliness. Based on a previous study,^46^ participants were categorized into three groups: “not lonely” (3 points), “lonely tendency” (4–5 points), and “lonely” (≥6 points).

### Covariates

The covariates included age, gender, living arrangement, marital status, educational attainment, household equivalent income, employment status, body mass index (BMI), illnesses currently under treatment, instrumental ADL (IADL) performance, drinking, smoking, physical activities, and residential area.

Age was categorized as “65–69,” “70–74,” “75–79,” “80–84”, and “≥85” years. Living arrangement was dichotomized as “living together” and “living alone.” Marital status was categorized as “married,” “divorced/separated,” and “never married.” Educational attainment was categorized as “<10,” “10–12”, and “≥13” years. Household equivalent income (million Japanese yen [JPY]) was calculated by dividing the income of each household by the square root of the household size (family member number) and was categorized as “low” (<2.00), “middle” (2.00–3.99), and “high” (≥4.00). Employment status was classified as “employed” and “not employed.” BMI (kg/m^2^) was categorized as “<18.5,” “18.5–24.9,” and “≥25.0.” Regarding the illnesses currently under treatment, musculoskeletal illness, fractures and injuries, and depressive disorder were assessed (“yes” or “no,” respectively). IADL performance was assessed using a five-item subscale,^47^ and participants with difficulty on ≥1 item were labeled as “with difficulty,” while others as “without difficulty.” Drinking and smoking were dichotomized as “current” and “past/never,” respectively. Physical activities were measured using a three-item subscale,^48^ and divided into tertiles according to calculated total METs/day (“low,” “middle,” and “high”). Residential area was assessed based on population density (people/km^2^) and classified as urban and suburban (≥1,000) and rural (<1,000).

### Statistical analysis

First, the descriptive statistics of participants’ characteristics were presented. Second, the prevalence of chronic low back pain according to social isolation and loneliness was calculated. Third, to examine the association of social isolation and loneliness with chronic low back pain, we conducted a multivariable Poisson regression analysis and obtained the prevalence ratios (PRs) and 95% confidence intervals (CIs) for chronic low back pain. To avoid overestimating the PRs, Poisson regression with robust variance was used, which can be regarded as a frequently occurring outcome.^49^ We conducted four analytical models. Model 1 was adjusted for age and gender. Model 2 was adjusted for the covariates examined (social isolation and loneliness were separately introduced into the analytical model). Model 3 was adjusted for the covariates and simultaneously introduced social isolation and loneliness. Model 4 was added to model 3, the interaction term of social isolation and loneliness. Fourth, we estimated each PR using nine groups with a combination of social isolation and loneliness, with a common reference group consisting of participants who reported neither isolation nor loneliness. To assess the validity of including both social isolation and loneliness simultaneously, Spearman’s rank correlation coefficient was calculated, showing a weak correlation (*r* = 0.215).

Sensitivity analyses were performed with similar analytical models, substituting the outcome variables with middle/severe chronic low back pain or chronic low back pain with disability in activities.

To examine potential differences in the association depending on the participants’ background, such as age, gender, and socioeconomic status, we conducted stratified analyses by the following variables: age (pre-old: 65–74 or old: ≥75 years),^50^ gender, educational attainment (low: <13 or high: ≥13 years), and household equivalent income (low: <2.00 or middle/high: ≥2.00 million JPY).

To mitigate potential bias due to missing information, we conducted the multiple imputation approach under the missing at random assumption, generated 20 imputed datasets using the multiple imputation by chained equations procedure, and pooled the results using the standard Rubin’s rule.^51^ We also conducted a complete-case analysis.

The significance level was set at 0.05. We used R software (Version 4.2.2 for Windows; R Foundation for Statistical Computing, Vienna, Austria) for all statistical analyses.

## RESULTS

Data from 21,463 participants were analyzed. Table 1 shows the participants’ characteristics. The mean age was 74.4 (standard deviation, 6.2) years; 51.5% were females. Chronic low back pain was experienced by 2,707 participants (12.6%). Those with chronic low back pain were more likely to be older, not married, less educated, have lower economic status, not employed, have higher BMI, have musculoskeletal disorders, fractures and injuries, and depressive disorder, have IADL difficulty, and have less physical activity. The prevalence of chronic low back pain did not change regardless of social isolation status but increased markedly in line with the level of loneliness.

**Table 1.** The characteristics of the participants

		Overall	Having chronic low back pain^a^
		(*n* = 21,463)	(*n* = 2,707)

		*n* (%)	Prevalence^b^, *n* (%)
Age, years	65–69	5,385 (25.1)	586 (10.9)
	70–74	6,346 (29.6)	718 (11.3)
	75–79	5,226 (24.3)	731 (14.0)
	80–84	3,041 (14.2)	444 (14.6)
	≥85	1,465 (6.8)	228 (15.6)
Gender	Male	10,399 (48.5)	1,364 (13.1)
	Female	11,064 (51.5)	1,343 (12.1)
Living arrangement	Living together	17,140 (79.9)	2,147 (12.5)
	Living alone	3,144 (14.7)	401 (12.8)
	Missing	1,179 (5.5)	159 (13.5)
Marital status	Married	15,639 (72.9)	1,928 (12.3)
	Divorced/separated	4,780 (22.3)	641 (13.4)
	Never married	649 (3.0)	87 (13.4)
	Other	113 (0.5)	16 (14.2)
	Missing	282 (1.3)	35 (12.4)
Educational attainment	<10	5,077 (23.7)	755 (14.9)
	10–12	9,219 (43.0)	1,145 (12.4)
	≥13	6,671 (31.1)	745 (11.2)
	Missing	496 (2.3)	62 (12.5)
Household equivalent income	Low	9,026 (42.1)	1,236 (13.7)
	Middle	4,692 (21.9)	557 (11.9)
	High	5,087 (23.7)	598 (11.8)
	Missing	2,658 (12.4)	316 (11.9)
Employment status	Not employed	13,463 (62.7)	1,756 (13.0)
	Employed	6,154 (28.7)	729 (11.8)
	Missing	1,846 (8.6)	222 (12.0)
BMI, kg/m^2^	<18.5	1,285 (6.0)	134 (10.4)
	18.5–24.9	14,358 (66.9)	1,693 (11.8)
	≥25.0	5,042 (23.5)	802 (15.9)
	Missing	778 (3.6)	78 (10.0)
Musculoskeletal illness	No	18,512 (86.3)	2,061 (11.1)
	Yes	2,135 (9.9)	576 (27.0)
	Missing	816 (3.8)	70 (8.6)
Fractures and injuries	No	20,279 (94.5)	2,546 (12.6)
	Yes	368 (1.7)	91 (24.7)
	Missing	816 (3.8)	70 (8.6)
Depressive disorder	No	20,491 (95.5)	2,611 (12.7)
	Yes	156 (0.7)	26 (16.7)
	Missing	816 (3.8)	70 (8.6)
IADL performance	Not difficulty	19,444 (90.6)	2,398 (12.3)
	Difficulty	1,472 (6.9)	243 (16.5)
	Missing	547 (2.5)	66 (12.1)
Smoking	Never/past	18,935 (88.2)	2,392 (12.6)
	Current	2,196 (10.2)	274 (12.5)
	Missing	332 (1.5)	41 (12.3)
Drinking	Never/past	11,783 (54.9)	1,465 (12.4)
	Current	8,909 (41.5)	1,118 (12.5)
	Missing	771 (3.6)	124 (16.1)
Physical activities	Low	7,286 (33.9)	1,021 (14.0)
	Middle	6,903 (32.2)	819 (11.9)
	High	6,257 (29.2)	751 (12.0)
	Missing	1,017 (4.7)	116 (11.4)
Residential area	Urban or suburban	14,340 (66.8)	1,770 (12.3)
	Rural	7,113 (33.1)	934 (13.1)
	Missing	10 (0.05)	3 (30.0)
Social isolation	Not isolated	9,771 (45.5)	1,185 (12.1)
	Isolation tendency	6,362 (29.6)	545 (13.0)
	Isolated	3,792 (17.7)	783 (13.1)
	Missing	1,538 (7.2)	194 (12.6)
Loneliness	Not lonely	12,200 (56.8)	1,345 (11.0)
	Lonely tendency	5,376 (25.0)	727 (13.5)
	Lonely	2,880 (13.4)	510 (17.7)
	Missing	1,007 (4.7)	125 (12.4)

BMI, body mass index; IADL, instrumental activities of daily living.

^a^Missing value: *n* = 2,098.

^b^Prevalent cases (percentage) for each category.

Table 2 shows the prevalence of chronic low back pain in nine groups of participants with different combinations of social isolation and loneliness. Regardless of the social isolation status, the prevalence of chronic low back pain was higher as the level of loneliness increased. For those with “not lonely” or “lonely tendency,” the prevalence remained almost unchanged as the level of social isolation increased. For those with “lonely,” the prevalence was higher as the level of social isolation status increased. Those without social isolation and loneliness had a 12.1% prevalence of chronic low back pain, while those with both had the highest prevalence at 21.6%.

**Table 2.** The prevalence of chronic low back pain in each group of social isolation and loneliness

		Loneliness		

		Not lonely	Lonely tendency	Lonely
Social isolation	Not isolated	737/5,329 (12.1%)	293/1,671 (14.9%)	135/656 (17.1%)
	Isolated tendency	369/3,150 (11.7%)	243/1,683 (14.4%)	179/924 (19.4%)
	Isolated	173/1,491 (11.6%)	162/1,136 (14.3%)	179/829 (21.6%)

Missing values: social isolation, *n* = 1,538; loneliness, *n* = 1,007; chronic low back pain, *n* = 2,098.

The data shows the number of individuals with chronic low back pain/number of individuals in the group (% chronic low back pain).

Table 3 shows the association of social isolation and loneliness with chronic low back pain, based on multivariable Poisson regression analysis. In model 2 with multivariable adjustment, social isolation was not significantly associated with the likelihood of chronic low back pain (compared with “not isolated,” “isolated tendency”: PR 0.99; 95% CI, 0.91–1.08; “isolated”: PR 1.05; 95% CI, 0.95–1.17), whereas loneliness demonstrated a statistically significant association (compared with “not lonely,” “lonely tendency”: PR 1.14; 95% CI, 1.04–1.24; “lonely”: PR 1.40; 95% CI, 1.27–1.53). These associations did not change substantially in model 3 with the simultaneous inclusion of social isolation and loneliness in the analytical model (compared with “not isolated,” “isolated tendency”: PR 0.96; 95% CI, 0.88–1.05; “isolated”: PR 0.99; 95% CI, 0.89–1.10; compared with “not lonely,” “lonely tendency”: PR 1.14; 95% CI, 1.05–1.25; “lonely”: PR 1.40; 95% CI, 1.27–1.54). Furthermore, social isolation and loneliness showed a significant positive interaction for the likelihood of chronic low back pain (model 4: “isolated” × “lonely,” PR 1.34; 95% CI, 1.05–1.72). Complete-case analysis showed almost the same tendencies of results (eTable 1).

**Table 3.** Association of social isolation and loneliness with chronic low back pain, multivariable Poisson regression analysis with multiple imputation approach

		Model 1		Model 2		Model 3		Model 4	
			
		Age- and gender-adjusted		Multivariable-adjusted		Multivariable-adjusted		The interaction term included^a^	
			
		PR (95% CI)	*P*-value	PR (95% CI)	*P*-value	PR (95% CI)	*P*-value	PR (95% CI)	*P*-value
Social isolation	Not isolated	1.00		1.00		1.00		1.00	
	Isolated tendency	1.02 (0.94–1.11)	0.661	0.99 (0.91–1.08)	0.889	0.96 (0.88–1.05)	0.357	0.93 (0.83–1.04)	0.198
	Isolated	1.10 (1.00–1.22)	0.057	1.05 (0.95–1.17)	0.335	0.99 (0.89–1.10)	0.824	0.92 (0.79–1.07)	0.282
Loneliness	Not lonely	1.00		1.00		1.00		1.00	
	Lonely tendency	1.20 (1.09–1.30)	<0.001	1.14 (1.04–1.24)	0.004	1.14 (1.05–1.25)	0.003	1.13 (0.99–1.29)	0.065
	Lonely	1.55 (1.41–1.70)	<0.001	1.40 (1.27–1.53)	<0.001	1.40 (1.27–1.54)	<0.001	1.20 (1.02–1.42)	0.029
Social isolation × Loneliness	Isolated tendency × lonely tendency							1.03 (0.84–1.27)	0.755
	Isolated × lonely tendency							1.03 (0.82–1.30)	0.810
	Isolated tendency × lonely							1.21 (0.95–1.53)	0.119
	Isolated × lonely							1.34 (1.05–1.72)	0.021

CI, confidence interval; PR, prevalence ratio.

In models 1 and 2, social isolation and loneliness were separately introduced into the analytical model, while in models 3 and 4, they were simultaneously introduced into the model.

Models 2, 3, and 4 were adjusted by age, gender, living arrangement, marital status, educational attainment, household equivalent income, employment status, body mass index, musculoskeletal illness, fractures and injuries, depressive disorders, instrumental activities of daily living performance, drinking, smoking, physical activities, and residential area.

^a^The interaction term (social isolation × loneliness) was introduced into the analytical model.

The sensitivity analyses, replacing outcome variables with moderate/severe-intensity chronic low back pain or with chronic low back pain with disability in activities, indicated that the results were broadly similar to the main analyses (eTable 2).

The stratified analyses by age (eTable 3) showed that loneliness was associated with chronic low back pain regardless of age; the interaction effect between social isolation and loneliness for chronic low back pain was substantial in the pre-old age group (model 4). Additionally, regardless of gender, loneliness showed an association with chronic low back pain (eTable 4), and in females, the interaction between social isolation and loneliness with this condition was found (model 4). In the stratification by socioeconomic factors (eTable 5), the associations of social isolation and loneliness with chronic low back pain were almost similar to the main analysis, whereas the interaction effect was pronounced in the lower socioeconomic strata (model 4).

**Figure 1.**
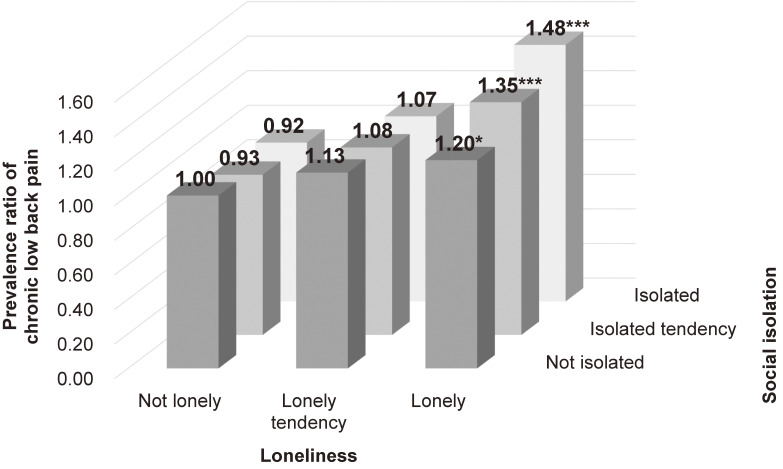
Prevalence ratio of chronic low back pain by the levels of combination between social isolation and loneliness. The bars show the prevalence ratios (PRs) of chronic low back pain of each group, with the group of participants who were neither isolated nor lonely taken as a common reference. PRs were adjusted for age, gender, living arrangement, marital status, educational attainment, household equivalent income, employment status, body mass index, musculoskeletal illness, fractures and injuries, depressive disorders, instrumental activities of daily living performance, drinking, smoking, physical activities, and residential area. ^*^, *P* < 0.05; ^***^, *P* < 0.001.

Figure 1 shows the PRs of chronic low back pain for each group of social isolation and loneliness, with the group of participants who were neither isolated nor lonely treated as a common reference. Regardless of social isolation status, the increased level of loneliness had higher PRs. Meanwhile, the increased social isolation had higher PRs only in those with “lonely” status. Participants who reported being both isolated and lonely were more likely to have chronic low back pain (PR 1.48; 95% CI, 1.28–1.72).

## DISCUSSION

This study of older adults indicated that loneliness was significantly associated with a higher likelihood of chronic low back pain, while it did not find an association of social isolation with this condition. Additionally, a positive interaction of social isolation and loneliness for chronic low back pain was found.

The participants who felt lonely were more likely to experience chronic low back pain; however, there was no association between social isolation and this condition. Previous studies have shown seemingly contradictory results: loneliness has been reported to be linked to low back pain,^33^ and contrastingly social isolation has not.^36^ However, considering the differences that social isolation is an objective state of lack of quantitative relationships with others, while loneliness is considered to be a subjective expression of negative feelings of missing relationships,^18^ our results are not in contradiction with the results of previous studies on social isolation and loneliness. Given previous findings that psychological distress and negative emotions were associated with the chronicity of pain,^9^^,^^10^ and that social exclusion, which is characterized by loneliness, shares with physical pain in which some brain circuity activated,^52^ it seems rational that this study found a significant association between loneliness and chronic low back pain. Our findings suggest that poor social relationships may be an important component of the social aspects in the biopsychosocial model of low back pain. Prior research has shown that people with chronic pain are more likely to lack social connectedness, so it is necessary to carefully note the bi-directionality and causality of these relationships.

This study found the multiplicative interactions of loneliness and social isolation for chronic low back pain. Although social isolation, by itself, was not associated with chronic low back pain, when combined with severe loneliness, it increased the likelihood of chronic low back pain. Accordingly, loneliness and social isolation may both be targeted in a social factor approach for addressing chronic low back pain. A serious lack of social connections activates physiological mechanisms, such as increased inflammatory response^21^; psychological mechanisms, such as enhanced anxiety and stress^53^^,^^54^; and social pathways, such as limited access to pain management and social support,^55^^,^^56^ possibly leading to the chronicity of low back pain. Social isolation is a lack of contact with others, which enhances the social aspects of pain, such as avoiding pain treatment and help from others, and might synergistically drive the pain worsened by loneliness. Hence, being socially isolated and experiencing further high levels of loneliness might constitute a difficult situation for an individual’s psychosocial well-being. However, the results did not show such associations in individuals who experienced social isolation but were not lonely; persons with little social contact with others but satisfaction with their social relationships might have low adverse health risks. Although the potential mechanisms and the influences of both social isolation and loneliness on chronic low back pain require further investigations, more attention may be needed to be paid toward the social contexts of individuals with low back pain to help improve their prognosis.

The stratified analysis showed broadly similar trends to the main analysis, whereas the combined effects of social isolation and loneliness for chronic low back pain were more substantial for pre-old age groups, females, and those in lower socioeconomic strata. Given the social-emotional selectivity theory,^57^ people in older age optimize their social networks to their satisfaction and do not expand the networks. Therefore, the adverse effects of social deficits may be somewhat modest in old age and the synergistic effects of social isolation and loneliness might be more pronounced in pre-old age. Additionally, as females spend more time in their community area than males,^58^ they tend to be more closely involved in their neighborhood social networks; nevertheless, females who are socially isolated and feel lonely may be more socially excluded. This indicates a severe psychosocial situation, which may be causing more stress and leading to chronic back pain. Meanwhile, for groups with low socioeconomic status, the conditions of having both isolation and loneliness may be severe social deprivation, which may lead to worse low back pain. Accordingly, the impact of social isolation and loneliness on low back pain might vary depending on the individual’s context, requiring the identification of social backgrounds likely to worsen it.

This study had several limitations. First, the cross-sectional study design could not determine causality, warranting further investigations using longitudinal data. Second, chronic low back pain was assessed through self-reported questionnaires rather than clinical diagnosis, which could have resulted in misclassification. However, several sensitivity analyses for chronic low back pain outcomes showed almost similar results, supporting the robustness of this study’s results. Third, this study was based on data from older adults without disabilities because this study included those who did not receive LTCI benefits. Further research is needed on the transportability of the results to individuals with disabilities and living limitations. Finally, it is unclear whether the study participants are representative of the entire Japanese population, which limited the generalizability of the results.

In conclusion, loneliness had a significant association with a higher likelihood of chronic low back pain among older adults; furthermore, those with both social isolation and loneliness were most likely to experience this condition. Our findings provided additional evidence for a social domain approach to chronic low back pain.

## SUPPLEMENTARY MATERIAL

eFigure 1. Illustration of the range of the low back

eTable 1. Association of social isolation and loneliness with chronic low back pain, multivariable Poisson regression analysis with complete case analysis (n = 13,194)

eTable 2. Association of social isolation and loneliness with severe/moderate chronic low back pain or chronic low back pain with disability in activities, multivariable Poisson regression analysis with multiple imputation approach

eTable 3. The stratified analysis by age on association of social isolation and loneliness with chronic low back pain, multivariable Poisson regression analysis with multiple imputation approach

eTable 4. The stratified analysis by gender on association of social isolation and loneliness with chronic low back pain, multivariable Poisson regression analysis with multiple imputation approach

eTable 5. The stratified analysis by educational attainment or economic status on association of social isolation and loneliness with chronic low back pain, multivariable Poisson regression analysis with multiple imputation approach
