# Supplementary material for: Association of Social Isolation and Loneliness With Chronic Low Back Pain Among Older Adults: A Cross-sectional Study From Japan Gerontological Evaluation Study (JAGES)
Source: J Epidemiol. 2024 Jun 5;34(6):270–7. doi: 10.2188/jea.JE20230127 (PMC11078594; doi:10.2188/jea.JE20230127)
Supplement: Supplementary file 1 [file je-34-270-s001.pdf]

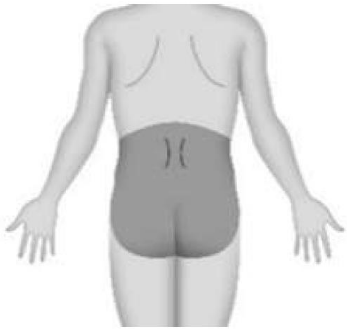

eFigure 1. Illustration of the range of the low back

eTable 1. Association of social isolation and loneliness with chronic low back pain, multivariable Poisson regression analysis with complete case analysis (n=13,194)

		Model 1		Model 2		Model 3		Model 4	
		Age- and gender-adjusted		Multivariable-adjusted		Multivariable-adjusted		The interaction term included[a]	
		PR (95% CI)	*P*-value	PR (95% CI)	*P*-value	PR (95% CI)	*P*-value	PR (95% CI)	*P*-value
Social isolation	Not isolated	1.00		1.00		1.00		1.00	
	Isolated tendency	1.02 (0.94–1.11)	0.565	1.01 (0.91–1.11)	0.915	0.97 (0.87–1.07)	0.503	0.88 (0.77–1.02)	0.090
	Isolated	1.11 (1.01–1.22)	0.034	1.07 (0.95–1.2)	0.274	0.99 (0.88–1.12)	0.908	0.92 (0.76–1.11)	0.382
Loneliness	Not lonely	1.00		1.00		1.00		1.00	
	Lonely tendency	1.23 (1.13–1.34)	<0.001	1.20 (1.09–1.33)	<0.001	1.20 (1.09–1.34)	<0.001	1.17 (1.01–1.36)	0.036
	Lonely	1.61 (1.46–1.77)	<0.001	1.42 (1.26–1.60)	<0.001	1.45 (1.28–1.64)	<0.001	1.13 (0.91–1.40)	0.266
Social isolation × Loneliness	Isolated tendency × lonely tendency							1.11 (0.88–1.40)	0.392
	Isolated × lonely tendency							1.02 (0.77–1.35)	0.902
	Isolated tendency × lonely							1.46 (1.09–1.96)	0.012
	Isolated × lonely							1.46 (1.07–2.00)	0.018

CI, confidence interval; PR, prevalence ratio.

In models 1 and 2, social isolation and loneliness were separately introduced into the analytical model, while in models 3 and 4, they were simultaneously introduced into the model.

Model 2, 3, and 4 were adjusted by age, gender, living arrangement, marital status, educational attainment, household equivalent income, employment status, body mass index, musculoskeletal illness, fractures and injuries, depressive disorders, instrumental activities of daily living performance, drinking, smoking, physical activities, and residential area.

[a]The interaction term (social isolation × loneliness) was introduced into the analytical model.

eTable 2. Association of social isolation and loneliness with severe/moderate chronic low back pain or chronic low back pain with disability in activities, multivariable Poisson regression analysis with multiple imputation approach

		Model 1		Model 2		Model 3		Model 4	
		Age- and gender-adjusted		Multivariable-adjusted		Multivariable-adjusted		The interaction term included[a]	
		PR (95% CI)	*P*-value	PR (95% CI)	*P*-value	PR (95% CI)	*P*-value	PR (95% CI)	*P*-value
**Moderate/severe chronic low back pain[b]**									
Social isolation	Not isolated	1.00		1.00		1.00		1.00	
	Isolated tendency	1.06 (0.99–1.14)	0.081	1.04 (0.97–1.12)	0.282	0.99 (0.93–1.07)	0.880	0.96 (0.87–1.07)	0.483
	Isolated	1.08 (0.99–1.17)	0.081	1.03 (0.94–1.12)	0.561	0.95 (0.87–1.03)	0.205	0.84 (0.73–0.96)	0.011
Loneliness	Not lonely	1.00		1.00		1.00		1.00	
	Lonely tendency	1.28 (1.19–1.37)	<0.001	1.24 (1.15–1.33)	<0.001	1.24 (1.16–1.33)	<0.001	1.18 (1.06–1.30)	0.002
	Lonely	1.64 (1.52–1.77)	<0.001	1.49 (1.38–1.61)	<0.001	1.51 (1.39–1.63)	<0.001	1.38 (1.20–1.58)	<0.001
Social isolation × Loneliness	Isolated tendency × lonely tendency							1.05 (0.90–1.24)	0.518
	Isolated × lonely tendency							1.23 (1.02–1.49)	0.028
	Isolated tendency × lonely							1.12 (0.92–1.36)	0.256
	Isolated × lonely							1.25 (1.01–1.54)	0.040
**Chronic low back pain with disability in activities[b]**									
Social isolation	Not isolated	1.00		1.00		1.00		1.00	
	Isolated tendency	1.09 (1.01–1.18)	0.025	1.05 (0.97–1.14)	0.230	0.98 (0.90–1.06)	0.596	0.99 (0.89–1.11)	0.883
	Isolated	1.13 (1.03–1.23)	0.007	1.05 (0.96–1.15)	0.270	0.93 (0.85–1.02)	0.113	0.80 (0.68–0.94)	0.007
Loneliness	Not lonely	1.00		1.00		1.00		1.00	
	Lonely tendency	1.47 (1.36–1.59)	<0.001	1.39 (1.29–1.50)	<0.001	1.40 (1.30–1.51)	<0.001	1.37 (1.22–1.53)	<0.001
	Lonely	2.05 (1.88–2.23)	<0.001	1.83 (1.68–2.00)	<0.001	1.86 (1.70–2.03)	<0.001	1.75 (1.53–2.02)	<0.001
Social isolation × Loneliness	Isolated tendency × lonely tendency							0.97 (0.81–1.16)	0.744

Isolated × lonely tendency	1.24 (0.99–1.55)	0.062
Isolated tendency × lonely	1.01 (0.83–1.24)	0.901
Isolated × lonely	1.29 (1.02–1.63)	0.034

CI, confidence interval; PR, prevalence ratio.

In models 1 and 2, social isolation and loneliness were separately introduced into the analytical model, while in models 3 and 4, they were simultaneously introduced into the model.

Models 2, 3, and 4 were adjusted by age, gender, living arrangement, marital status, educational attainment, household equivalent income, employment status, body mass index, musculoskeletal illness, fractures and injuries, depressive disorders, instrumental activities of daily living performance, drinking, smoking, physical activities, and residential area.

[a]The interaction term (social isolation × loneliness) was introduced into the analytical model.

[b]The number of participants with moderate/severe chronic low back pain and chronic low back pain with disability in activities was 1,817 (8.5%) and 1,902 (8.9%), respectively.

eTable 3. The stratified analysis by age on association of social isolation and loneliness with chronic low back pain, multivariable Poisson regression analysis with multiple imputation approach

		Model 1		Model 2		Model 3		Model 4	
		Age- and gender-adjusted		Multivariable-adjusted		Multivariable-adjusted		The interaction term included[a]	
		PR (95% CI)	*P*-value	PR (95% CI)	*P*-value	PR (95% CI)	*P*-value	PR (95% CI)	*P*-value
**Aged 65 to 74 years (n=11,731)**									
Social isolation	Not isolated	1.00		1.00		1.00		1.00	
	Isolated tendency	1.01 (0.89–1.13)	0.924	0.97 (0.87–1.10)	0.677	0.94 (0.83–1.05)	0.276	0.91 (0.77–1.08)	0.278
	Isolated	1.05 (0.92–1.21)	0.479	0.99 (0.86–1.14)	0.840	0.91 (0.79–1.06)	0.225	0.82 (0.65–1.03)	0.092
Loneliness	Not lonely	1.00		1.00		1.00		1.00	
	Lonely tendency	1.20 (1.06–1.35)	0.003	1.14 (1.01–1.29)	0.029	1.16 (1.02–1.31)	0.019	1.17 (0.98–1.40)	0.075
	Lonely	1.65 (1.44–1.88)	<0.001	1.46 (1.27–1.67)	<0.001	1.49 (1.29–1.71)	<0.001	1.21 (0.96–1.53)	0.104
Social isolation × Loneliness	Isolated tendency × lonely tendency							0.95 (0.71–1.27)	0.736
	Isolated × lonely tendency							1.07 (0.77–1.51)	0.677
	Isolated tendency × lonely							1.31 (0.94–1.83)	0.114
	Isolated × lonely							1.47 (1.01–2.13)	0.044
**Aged ≥75 years (n=9,732)**									
Social isolation	Not isolated	1.00		1.00		1.00		1.00	
	Isolated tendency	1.03 (0.92–1.16)	0.579	1.00 (0.89–1.13)	0.957	0.98 (0.86–1.10)	0.696	0.93 (0.80–1.09)	0.371
	Isolated	1.16 (1.02–1.33)	0.031	1.11 (0.97–1.27)	0.144	1.06 (0.92–1.21)	0.451	1.01 (0.82–1.23)	0.960
Loneliness	Not lonely	1.00		1.00		1.00		1.00	
	Lonely tendency	1.19 (1.06–1.34)	0.004	1.13 (1.01–1.27)	0.042	1.13 (1.00–1.27)	0.048	1.10 (0.91–1.31)	0.322
	Lonely	1.47 (1.30–1.67)	<0.001	1.33 (1.17–1.51)	<0.001	1.32 (1.16–1.50)	<0.001	1.17 (0.93–1.48)	0.189

Social isolation × Loneliness	Isolated tendency × lonely tendency	1.11 (0.86–1.44)	0.420
	Isolated × lonely tendency	0.99 (0.73–1.36)	0.966
	Isolated tendency × lonely	1.14 (0.83–1.55)	0.428
	Isolated × lonely	1.28 (0.90–1.81)	0.170

CI, confidence interval; PR, prevalence ratio.

In models 1 and 2, social isolation and loneliness were separately introduced into the analytical model, while in models 3 and 4, they were simultaneously introduced into the model.

Models 2, 3, and 4 were adjusted by living arrangement, marital status, educational attainment, household equivalent income, employment status, body mass index, musculoskeletal illness, fractures and injuries, depressive disorders, instrumental activities of daily living performance, drinking, smoking, physical activities, and residential area.

[a]The interaction term (social isolation × loneliness) was introduced into the analytical model.

eTable 4. The stratified analysis by gender on association of social isolation and loneliness with chronic low back pain, multivariable Poisson regression analysis with multiple imputation approach

		Model 1		Model 2		Model 3		Model 4	
		Age- and gender-adjusted		Multivariable-adjusted		Multivariable-adjusted		The interaction term included[a]	
		PR (95% CI)	*P*-value	PR (95% CI)	*P*-value	PR (95% CI)	*P*-value	PR (95% CI)	*P*-value
**Male (n=10,399)**									
Social isolation	Not isolated	1.00		1.00		1.00		1.00	
	Isolated tendency	0.97 (0.86–1.09)	0.570	0.96 (0.85–1.08)	0.466	0.92 (0.82–1.04)	0.203	0.91 (0.77–1.07)	0.240
	Isolated	1.06 (0.93–1.20)	0.400	1.02 (0.89–1.16)	0.789	0.95 (0.83–1.08)	0.419	0.98 (0.81–1.19)	0.839
Loneliness	Not lonely	1.00		1.00		1.00		1.00	
	Lonely tendency	1.17 (1.03–1.32)	0.015	1.13 (1.00–1.28)	0.056	1.14 (1.00–1.29)	0.046	1.23 (1.03–1.47)	0.021
	Lonely	1.62 (1.42–1.83)	<0.001	1.48 (1.30–1.69)	<0.001	1.50 (1.32–1.72)	<0.001	1.28 (1.01–1.64)	0.046
Social isolation × Loneliness	Isolated tendency × lonely tendency							0.90 (0.69–1.18)	0.454
	Isolated × lonely tendency							0.85 (0.63–1.14)	0.281
	Isolated tendency × lonely							1.32 (0.94–1.87)	0.110
	Isolated × lonely							1.14 (0.81–1.59)	0.446
**Female (n=11,064)**									
Social isolation	Not isolated	1.00		1.00		1.00		1.00	
	Isolated tendency	1.06 (0.95–1.19)	0.302	1.03 (0.92–1.15)	0.628	1.00 (0.89–1.12)	0.946	0.95 (0.81–1.11)	0.526
	Isolated	1.16 (1.00–1.35)	0.055	1.09 (0.94–1.27)	0.260	1.04 (0.89–1.21)	0.651	0.83 (0.64–1.07)	0.148
Loneliness	Not lonely	1.00		1.00		1.00		1.00	
	Lonely tendency	1.23 (1.09–1.38)	0.001	1.15 (1.02–1.30)	0.022	1.15 (1.02–1.30)	0.024	1.06 (0.89–1.27)	0.515
	Lonely	1.49 (1.30–1.70)	<0.001	1.32 (1.15–1.51)	<0.001	1.31 (1.14–1.51)	<0.001	1.15 (0.93–1.43)	0.206

Social isolation × Loneliness	Isolated tendency × lonely tendency	1.16 (0.88–1.53)	0.294
	Isolated × lonely tendency	1.33 (0.91–1.92)	0.138
	Isolated tendency × lonely	1.10 (0.81–1.50)	0.544
	Isolated × lonely	1.70 (1.16–2.51)	0.007

CI, confidence interval; PR, prevalence ratio.

In models 1 and 2, social isolation and loneliness were separately introduced into the analytical model, while in models 3 and 4, they were simultaneously introduced into the model.

Models 2, 3, and 4 were adjusted by age, living arrangement, marital status, educational attainment, household equivalent income, employment status, body mass index, musculoskeletal illness, fractures and injuries, depressive disorders, instrumental activities of daily living performance, drinking, smoking, and physical activities, and residential area.

[a]The interaction term (social isolation × loneliness) was introduced into the analytical model.

eTable 5. The stratified analysis by educational attainment or economic status on association of social isolation and loneliness with chronic low back pain, multivariable Poisson regression analysis with multiple imputation approach

		Model 1		Model 2		Model 3		Model 4	
		Age- and gender-adjusted		Multivariable-adjusted		Multivariable-adjusted		The interaction term included[a]	
		PR (95% CI)	*P*-value	PR (95% CI)	*P*-value	PR (95% CI)	*P*-value	PR (95% CI)	*P*-value
**Stratification by educational attainment**									
**Low (<13 years; n=14,296)**									
Social isolation	Not isolated	1.00		1.00		1.00		1.00	
	Isolated tendency	1.04 (0.94–1.15)	0.434	1.02 (0.92–1.12)	0.739	0.98 (0.89–1.09)	0.748	0.95 (0.83–1.09)	0.500
	Isolated	1.14 (1.01–1.27)	0.031	1.09 (0.97–1.22)	0.157	1.03 (0.91–1.15)	0.661	0.93 (0.78–1.11)	0.419
Loneliness	Not lonely	1.00		1.00		1.00		1.00	
	Lonely tendency	1.19 (1.08–1.32)	<0.001	1.14 (1.03–1.26)	0.013	1.14 (1.03–1.26)	0.014	1.13 (0.97–1.32)	0.123
	Lonely	1.48 (1.33–1.64)	<0.001	1.36 (1.23–1.52)	<0.001	1.36 (1.22–1.51)	<0.001	1.14 (0.94–1.39)	0.181
Social isolation × Loneliness	Isolated tendency × lonely tendency							1.01 (0.79–1.28)	0.965
	Isolated × lonely tendency							1.07 (0.82–1.40)	0.627
	Isolated tendency × lonely							1.23 (0.93–1.62)	0.141
	Isolated × lonely							1.41 (1.06–1.88)	0.019
**High (≥13 years; n=6,671)**									
Social isolation	Not isolated	1.00		1.00		1.00		1.00	
	Isolated tendency	0.95 (0.82–1.12)	0.561	0.94 (0.81–1.10)	0.462	0.91 (0.77–1.06)	0.222	0.86 (0.70–1.06)	0.155
	Isolated	0.98 (0.81–1.19)	0.850	0.96 (0.79–1.17)	0.678	0.88 (0.72–1.07)	0.210	0.90 (0.68–1.19)	0.456
Loneliness	Not lonely	1.00		1.00		1.00		1.00	
	Lonely tendency	1.19 (1.02–1.4)	0.031	1.14 (0.98–1.34)	0.099	1.16 (0.99–1.37)	0.070	1.14 (0.91–1.44)	0.246

	Lonely	1.70 (1.41–2.04)	<0.001	1.56 (1.29–1.89)	<0.001	1.61 (1.32–1.96)	<0.001	1.48 (1.07–2.05)	0.018
Social isolation × Loneliness	Isolated tendency × lonely tendency							1.12 (0.78–1.60)	0.536
	Isolated × lonely tendency							0.90 (0.59–1.39)	0.639
	Isolated tendency × lonely							1.15 (0.72–1.81)	0.561
	Isolated × lonely							1.11 (0.67–1.83)	0.680
**Stratification by economic status**									
**Low (n=9,026)**									
Social isolation	Not isolated	1.00		1.00		1.00		1.00	
	Isolated tendency	1.04 (0.93–1.17)	0.522	1.04 (0.92–1.17)	0.543	1.00 (0.89–1.13)	0.961	0.94 (0.80–1.10)	0.420
	Isolated	1.12 (0.99–1.27)	0.076	1.09 (0.96–1.24)	0.174	1.03 (0.90–1.17)	0.685	0.87 (0.70–1.07)	0.196
Loneliness	Not lonely	1.00		1.00		1.00		1.00	
	Lonely tendency	1.17 (1.04–1.32)	0.010	1.12 (0.99–1.26)	0.063	1.12 (0.99–1.26)	0.072	1.05 (0.86–1.28)	0.636
	Lonely	1.54 (1.37–1.74)	<0.001	1.40 (1.24–1.58)	<0.001	1.39 (1.23–1.57)	<0.001	1.14 (0.92–1.41)	0.240
Social isolation × Loneliness	Isolated tendency × lonely tendency							1.11 (0.84–1.47)	0.477
	Isolated × lonely tendency							1.22 (0.87–1.71)	0.260
	Isolated tendency × lonely							1.28 (0.93–1.75)	0.133
	Isolated × lonely							1.53 (1.10–2.13)	0.012
**Middle/high (n=9,026)**									
Social isolation	Not isolated	1.00		1.00		1.00		1.00	
	Isolated tendency	0.99 (0.87–1.11)	0.806	0.94 (0.83–1.06)	0.320	0.91 (0.80–1.02)	0.115	0.91 (0.77–1.07)	0.264
	Isolated	1.05 (0.90–1.22)	0.537	1.00 (0.86–1.17)	0.997	0.94 (0.80–1.10)	0.422	0.98 (0.78–1.23)	0.869

Loneliness	Not lonely	1.00		1.00		1.00		1.00	
	Lonely tendency	1.21 (1.07–1.37)	0.003	1.16 (1.02–1.32)	0.025	1.17 (1.03–1.33)	0.017	1.23 (1.04–1.46)	0.017
	Lonely	1.48 (1.27–1.71)	<0.001	1.39 (1.19–1.61)	<0.001	1.41 (1.21–1.65)	<0.001	1.32 (1.00–1.74)	0.049
Social isolation × Loneliness	Isolated tendency × lonely tendency							0.95 (0.71–1.26)	0.727
	Isolated × lonely tendency							0.84 (0.60–1.17)	0.292
	Isolated tendency × lonely							1.09 (0.74–1.60)	0.667
	Isolated × lonely							1.09 (0.71–1.66)	0.693

CI, confidence interval; PR, prevalence ratio.

In models 1 and 2, social isolation and loneliness were separately introduced into the analytical model, while in models 3 and 4, they were simultaneously introduced into the model.

Models 2, 3, and 4 were adjusted by age, living arrangement, marital status, educational attainment, household equivalent income, employment status, body mass index, musculoskeletal illness, fractures and injuries, depressive disorders, instrumental activities of daily living performance, drinking, smoking, physical activities, and residential area (educational attainment and household equivalent income were excluded as covariates for each stratified analysis).

[a]The interaction term (social isolation × loneliness) was introduced into the analytical model.
